# Editorial note: Sunbathing, a possible risk factor of murine typhus infection in Greece

**DOI:** 10.1371/journal.pntd.0014448

**Published:** 2026-06-22

**Authors:** The PLOS Neglected Tropical Diseases Editors

The *PLOS Neglected Tropical Diseases* Editors issue this notice to update the previously published Expression of Concern on this article [1,2].

Following the publication of the article and Expression of Concern [1,2], PLOS investigated concerns pertaining to the reported ethical approval and the article’s adherence to *PLOS Neglected Tropical Diseases*’ research ethics policies.

The Materials section in [1] reports that the study involved the use of serum samples of hospitalized patients and outpatients in Greece collected between January 2012 – December 2019, as well as access to patients’ clinical data and medical history. The ethics statement reported in this article states that “All collected data were anonymized in standardized forms according to the Ethic and Scientific Committee of the Hellenic Pasteur Institute under registration number EIP-GDPR-E01.01.”

A representative of the Aix-Marseille Université Ethics Committee stated that the institutional investigation into the ethics concerns concluded this article meets ethical standards. They provided a copy of document N° EIP-GDPR-E01.01 for editorial review.

PLOS reviewed the documentation provided by the institution and concluded that the documents did not fully resolve the journal’s concerns. Specifically,

- Document N° EIP-GDPR-E01.01, issued by the Institut Pasteur Hellenique (referred to in this article [1] by its English name the Hellenic Pasteur Institute) on May 29, 2018, is not an ethics approval document, but a consent form for the processing of personal data for diagnostic purposes and patient tracing. Although the document states that the institute may retain, use, and process the personal data related to diagnosis for research purposes, the form appears to be a general consent form and does not contain any information to suggest that the study reported in this article [1] was reviewed and approved by an independent ethics body.
- Document N° EIP-GDPR-E01.01 does not appear to cover the full data collection period.

The corresponding author and the fourth author responded to the journal’s outstanding concerns stating that at the time of the manuscript submission, the Hellenic Pasteur Institute lacked an Ethics Committee, and therefore document N° EIP-GDPR-E01.01 was the only official certification available. The authors’ explanation appears to contradict the Ethics statement in the article, which suggests that the Hellenic Pasteur Institute had an Ethics and Scientific Committee.

PLOS has been unable to confirm with independent sources whether, under Greek legislation relevant at the time the study was conducted, this study would have required formal ethics approval, or whether this study would have been exempted from formal ethics approval. Therefore, the Expression of Concern stands.
